# Assessment of Patient Characteristics Influencing the Analgesic Effects of Ibuprofen Gargle After Mandibular Third Molar Extractions

**DOI:** 10.7759/cureus.57516

**Published:** 2024-04-03

**Authors:** Yasumasa Kakei, Takeshi Ioroi, Keiko Miyakoda, Takahiro Ito, Masahiko Kashin, Tatsuya Shirai, Takumi Hasegawa, Toshiyasu Sakane, Ikuko Yano, Masaya Akashi

**Affiliations:** 1 Oral and Maxillofacial Surgery, Kobe University Hospital, Kobe, JPN; 2 Pharmacy, Kobe University Hospital, Kobe, JPN; 3 Clinical and Translational Research Center, Kobe University Hospital, Kobe, JPN; 4 Oral and Maxillofacial Surgery, Kobe University Graduate School of Medicine, Kobe, JPN; 5 Pharmaceutical Technology, Kobe Pharmaceutical University, Kobe, JPN

**Keywords:** tooth extraction, postoperative pain, mouthwash, mandibular third molar, ibuprofen

## Abstract

Introduction

In our previous work, we investigated the analgesic effects of ibuprofen gargle after mandibular third molar extractions. However, a subsequent detailed review of individual patient data revealed variations in postoperative pain reduction among patients. Consequently, the present study was designed to conduct post-hoc subanalyses that identified factors contributing to variation in the analgesic response to ibuprofen gargle after third molar extractions.

Materials and methods

This study involved thirty-five Japanese patients from a prior randomized, double-blind, placebo-controlled, crossover study, which focused on the analgesic effects of ibuprofen gargle after mandibular third molar extractions. Participants were categorized as responders (n = 13) and non-responders (n = 22) based on the within-subject difference (ibuprofen-placebo, IP) of visual analog scale (VAS) changes. Baseline characteristics were compared, along with variables, such as age, sex, the reason for extraction, extraction site, Pell Gregory (space and depth) classification, Winter’s classification, surgeon’s experience, and surgery time. Baseline characteristics predicting responder status were examined using multivariate logistic regression.

Results

In the univariate analysis, variables such as age, sex, and baseline VAS scores with p-values <0.2 were evaluated using a stepwise approach. This analysis identified age (per -10 years) with an odds ratio of 4.163 (95% confidence interval (CI): 1.170-31.952, p = 0.0233) and sex (female) with an odds ratio of 9.977 (95% CI: 1.336-208.256, p = 0.0213) as significant predictors of responder status.

Conclusions

In young and female patients, ibuprofen gargle decreased postoperative pain after mandibular third molar extractions.

## Introduction

Extraction of the mandibular third molar is a common oral surgery procedure [1,2]. This procedure is invasive, particularly when bone removal and crown division is involved; it often leads to moderate-to-severe postoperative complications such as pain, swelling, and restricted mouth opening and swallowing, along with challenges in oral intake after surgery [2,3]. Post-extraction pain is a primary concern after dental extractions, often deterring patients from seeking treatment [1]. Nonsteroidal anti-inflammatory drugs (NSAIDs) and acetaminophen are standard analgesics for the management of post-extraction pain; NSAIDs, particularly acidic NSAIDs, are typically preferred for moderate pain, such as the pain associated with mandibular extractions [4]. For patients with gastrointestinal ulcers or aspirin-induced asthma, acetaminophen may serve as an alternative [4]. Various NSAIDs, including celecoxib [5,6], valdecoxib [7], ibuprofen [8], flurbiprofen [9], and opioid-containing medications such as oxycodone [10], have been closely evaluated to identify optimal analgesics for pain alleviation in mandibular third molar extractions. A systematic review by Weil et al. highlighted the safety and efficacy of oral paracetamol (acetaminophen) in postoperative pain management after embedded mandibular third molar extractions [11], whereas the Cochrane review by Bailey et al. highlighted the superiority of oral ibuprofen over oral paracetamol in this context [12].

When prescribing postoperative analgesics, clinicians must consider potential side effects [13]; opioids are primarily linked to respiratory depression, nausea, vomiting, and constipation, whereas NSAIDs are predominantly associated with gastrointestinal, renal, and hepatic complications, as well as platelet dysfunction [14]. Rindal et al. recently outlined a randomized controlled trial protocol to mitigate opioid-related issues, with a focus on interventions to reduce opioid prescriptions in favor of nonopioid alternatives [15]. Furthermore, topical analgesic application has emerged as a potentially safer approach to enhance postoperative pain relief [16]; studies have shown reduced side effects without decreases in analgesic efficacy [9,17,18].

Ibuprofen, introduced in the 1960s, is a potent inhibitor of prostaglandin synthesis that effectively mitigates fever, pain, and inflammation [19]. Despite its pharmacological activity against cyclooxygenase-1 and cyclooxygenase-2, systemic administration may cause adverse effects such as gastrointestinal and renal dysfunction. Nevertheless, numerous reviews and meta-analyses have confirmed ibuprofen’s efficacy and relatively low toxicity compared with other NSAIDs, for both adults and children [20-22]. We hypothesized that an oral ibuprofen gargle (0.6% or 1%) directly applied to the affected area could alleviate pain associated with oral mucositis [23]. Previous research indicated no significant safety concerns and suggested that some pain relief could be achieved in cases of chemotherapy- or chemoradiotherapy-induced oral mucositis [23].

Considering the rapid absorption of locally administered drugs in post-extraction wounds due to the loss of keratinized mucosa [24], we hypothesized that an ibuprofen mouthwash could serve as an efficient drug delivery system for targeting post-extraction pain while minimizing systemic effects. In this context, we conducted a single-center, placebo-controlled, double-blind, randomized crossover study to evaluate the efficacy of ibuprofen mouthwash [25,26]. The study failed to demonstrate a statistically significant effect on post-extraction pain relief, despite not observing any major safety issues, including increases in postoperative bleeding [26].

On the basis of these findings, and in anticipation of the insurance coverage trials that will begin in Japan in June 2024, this clinical study was meticulously designed to evaluate the differential effects of ibuprofen gargle on postoperative pain relief after mandibular third molar extractions. The primary goal of the study was the identification of patient characteristics that could predict an effective analgesic response to ibuprofen gargle. Consequently, the study aims to establish a definitive relationship between its objectives and the hypothesis that specific baseline characteristics, including age, sex, and initial pain levels, significantly influence the efficacy of ibuprofen gargle as a postoperative analgesic treatment.

## Materials and methods

Data source and study population

This study was conducted as a substudy of an exploratory clinical trial that evaluated the clinical effects of administering ibuprofen gargle to patients after mandibular third molar extractions. The design and main results of the exploratory clinical trial have been previously reported [26]. The primary objective of this substudy was to examine specific patient subgroups within the same cohort, particularly responders and non-responders, to determine whether baseline characteristics (e.g., age and sex) influenced the efficacy of the study treatment. The study protocol complied with the Declaration of Helsinki and was approved by the ethics committee of our hospital (C200024, date of approval: March 23, 2021). Written informed consent was obtained from all patients.

Briefly, the original study was an investigator-initiated, placebo-controlled, double-blind, randomized crossover, single-center clinical trial in which the study intervention comprised gargling 0.6% ibuprofen daily for five days [25]. Patients were enrolled after providing written informed consent to participate. The main exclusion criteria were peptic ulcers, a history of hypersensitivity to any component of the ibuprofen gargle, impaired cardiac function or clinically significant heart disease, and aspirin-induced asthma. A visual analog scale (VAS) was used to measure pain before and at five minutes and 15 minutes after gargling, daily for up to one week [25].

**Figure 1:**
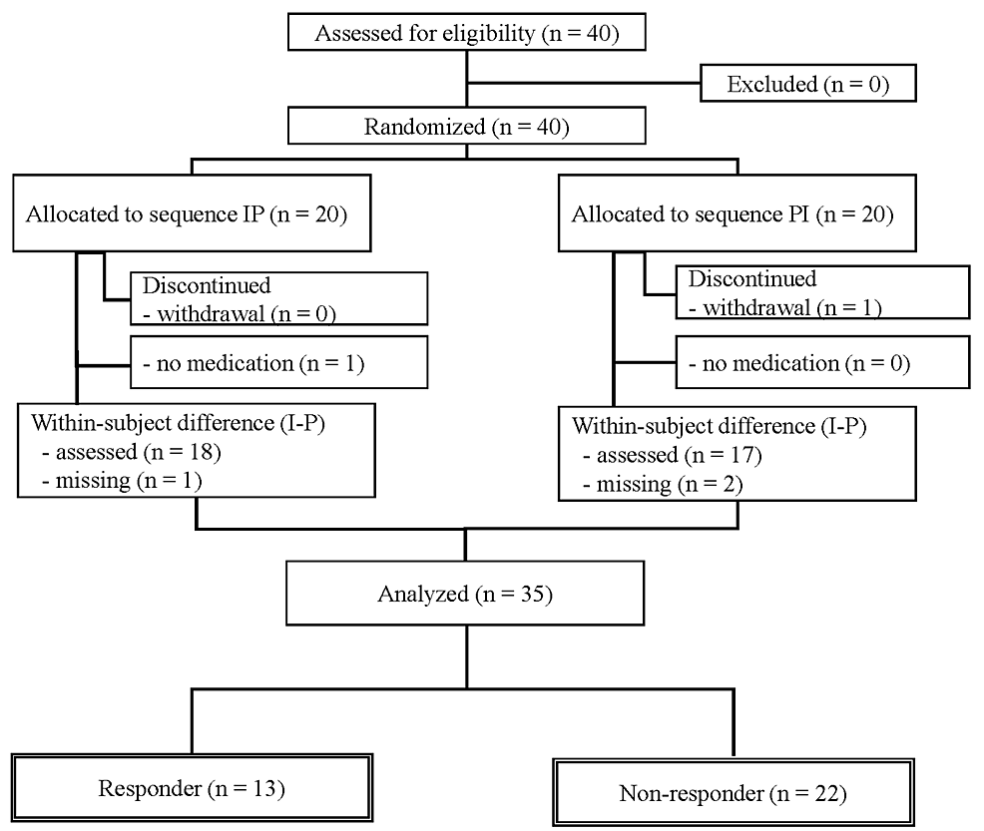
Patient flow chart for the assessment of patient characteristics associated with the efficacy of ibuprofen gargle treatment. I: ibuprofen, P: placebo.

From 7 June 2021 to 26 May 2022, 40 patients were enrolled [26]. However, one patient withdrew informed consent, and four patients for whom VAS scores could not be obtained on postoperative day (POD) 1 and POD 2 were excluded [26]. Therefore, the study population in this substudy comprised 35 patients (Figure 1).

Patient characteristics associated with treatment efficacy

In the original study, patients were randomized at a 1:1 ratio into two groups: ibuprofen-placebo (IP) and placebo-ibuprofen (PI). On POD 1, ibuprofen gargle administration was initiated in the IP group, whereas the PI group began using a placebo gargle. On POD 2, the IP group switched to a placebo gargle, and the PI group switched to an ibuprofen gargle. On PODs 3-5, both groups were prescribed an ibuprofen gargle. Approximately 10 mL of gargle solution was dispensed into a measuring cup and retained in the mouth in contact with the affected area for at least 30 seconds (preferably one minute), then discarded [25].

The primary endpoint was the change in pain intensity, as measured by the VAS, within each patient before and five minutes after the first use of either the ibuprofen or placebo gargle on PODs 1 and 2 (ΔVAS5_ibuprofen−ΔVAS5_placebo). This change is denoted as ΔVAS5, where 'Δ' represents the difference and '5' indicates the time in minutes after administration. Specifically, ΔVAS5_ibuprofen and ΔVAS5_placebo refer to changes in VAS scores after the use of ibuprofen and placebo gargles, respectively.

The equation ΔVAS5_ibuprofen−ΔVAS5_placebo calculates the difference in pain score changes between the ibuprofen and placebo treatments. A negative value indicates that the ibuprofen gargle led to a greater reduction in pain compared with the placebo gargle. For example, in our study, the within-patient changes in VAS5 for the IP and PI groups were 1.25 ± 12.0 and −5.26 ± 8.93 mm, respectively, demonstrating how pain levels changed after each treatment. The overall treatment effect of the ibuprofen gargle was calculated as −2.01 ± 10.62 mm (p = 0.246), suggesting that, on average, the ibuprofen gargle reduced pain slightly more than the placebo; however, this difference was not statistically significant. The treatment effect was determined by calculating the mean of within-patient changes in VAS scores for both the IP and PI groups, providing a measure of the ibuprofen gargle's mean effectiveness in pain reduction across the study population. Instead of performing the evaluation solely after five minutes, clinical acceptability was defined as the persistence of the effect after 15 minutes. A subanalysis of VAS changes was performed with a response of 10 mm or greater in the group of patients. Therefore, the responders defined within-patient difference (I-P) at five or 15 minutes as −10 or less in this study. Correlations of within-patient differences (I-P) at five and 15 minutes were also examined. After excluding patients with no VAS data for both POD 1 and 2, the characteristics of responders and non-responders were compared in this substudy.

**Figure 2:**
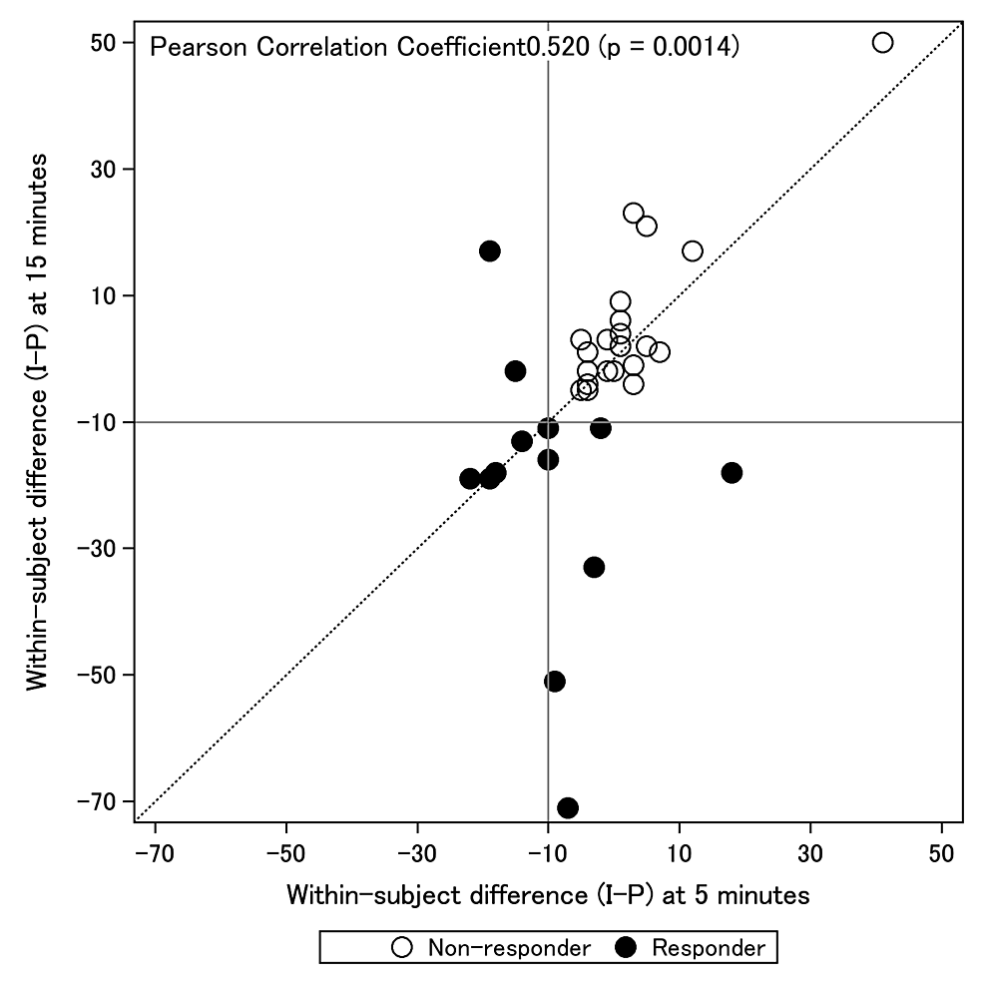
Correlation of within-subject difference (I-P) at five minutes with within-subject difference (I-P) at 15 minutes. I: ibuprofen, P: placebo.

As shown in Figure 2, the correlation of within-subject difference (I-P) at five and 15 minutes had a Pearson correlation coefficient of 0.520 (p = 0.0014).

Statistical analysis of endpoints

**Figure 3:**
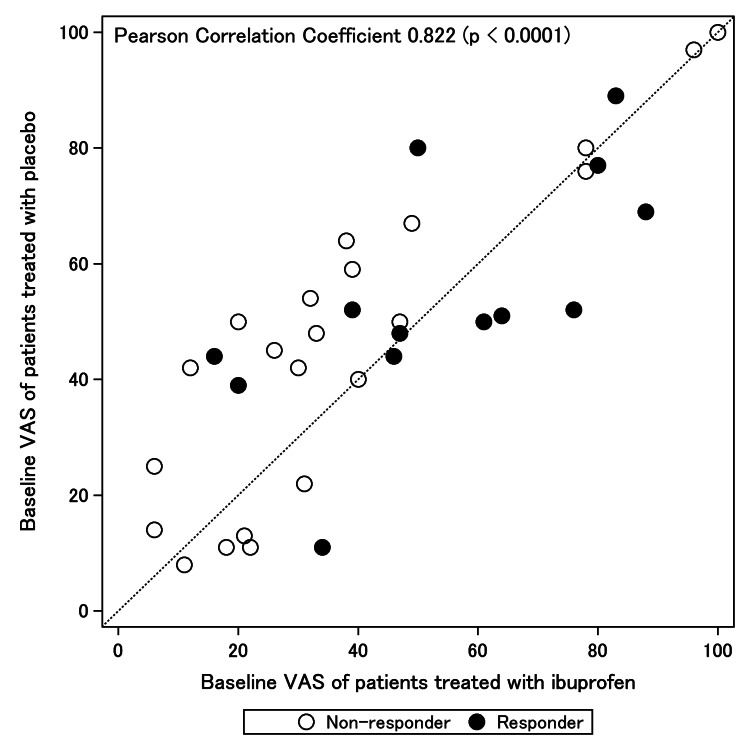
Correlation of the baseline visual analog scale (VAS) of ibuprofen treatment with the baseline VAS of the placebo. VAS: visual analog scale, I: ibuprofen, P: placebo.

The results are primarily presented as descriptive statistics because of the limited sample size. Continuous variables are expressed as means ± standard deviations (SDs), whereas categorical variables are represented as numbers (%). Baseline characteristics and variables were assessed using Welch’s t-test and Fisher’s exact test. Multivariate logistic regression analysis was performed with ‘responder’ as the dependent variable. This analysis utilized a stepwise approach, selecting variables with a p-value of <0.2 from the univariate analysis of baseline characteristics as candidate explanatory variables. Akaike’s information criterion (AIC) was used to guide the variable selection process. The stepwise approach determined the final model, which provided the odds ratios and 95% confidence intervals (CIs) for the variables. Because baseline VAS scores were highly correlated with both ibuprofen and placebo use (Pearson correlation coefficient of 0.822), as shown in Figure 3, we used the mean of both VAS scores. Data were analyzed using R version 4.2.1 software (R Core Team, Vienna, Austria).

## Results

Patient characteristics associated with the treatment efficacy

Of the 35 patients who received ibuprofen gargle, 13 were responders and 22 were non-responders. The characteristics of responders and non-responders are shown in Table 1.

**Table 1:** Comparison of baseline characteristics between responders and non-responders to ibuprofen gargle treatment. VAS: visual analog scale, I: ibuprofen, P: placebo.

Characteristic		All (n = 35)	Responders (n = 13)^a^	Non-responders (n = 22)^a^	p-value
Age (years)		29.7 ± 8.8	25.2 ± 2.6	32.4 ± 10.2	0.0044^b^
Sex					
Male		12 (34.3%)	1 (12%)	11 (50. 0%)	0.0132^c^
Female		23 (65.7%)	12 (88%)	11 (50.0%)	
Reason for extraction					0.2591^c^
Pericoronitis		25 (71.4%)	11 (84.6%)	14 (63.6%)	
Other		10 (28.6%)	2 (15.4%)	8 (36.4%)	
Pell Gregory classification (distal_space)					0.2591^c^
Class I		3 (8.6%)	0 (0.0%)	3 (13.6%)	
Class II		32 (91.4%)	13 (100.0%)	19 (86.0%)	
Pell Gregory classification (depth)					0.7260^c^
Class A		23 (65.7%)	8 (61.5%)	15 (68.2%)	
Class B		12 (34.3%)	5 (38.5%)	7 (31.8%)	
Winter’s classification					0.4930^c^
Horizontal		13 (37.1%)	6 (46.2%)	7 (31.8%)	
Vertical		13 (37.1%)	3 (23.1%)	10 (45.5%)	
Mesioangular		6 (17.1%)	2 (15.4%)	4 (18.2%)	
Distoangular		3 (8.6%)	2 (15.4%)	1 (4.5%)	
Surgeon experience (years)		6.1 ± 1.6	6.0 ± 1.8	6.1 ± 1.5	0.8780^b^
Surgery time (min)		28.4 ± 10.2	29.2 ± 12.7	28.0 ± 8.7	0.7666^b^
Baseline VAS (mean of I and P)		46.6 ± 24.7	54.2 ± 20.0	42.1 ± 26.5	0.1353^b^
Treatment sequence					0.3053^c^
PI		17 (48.6%)	8 (61.5%)	9 (40.9%)	
IP		18 (51.4%)	5 (38.5%)	13 (59.1%)	
Within-subject difference (I-P) (VAS5)		−2.1 ± 11.5	−10.0 ± 10.5	2.5 ± 9.6	0.0017^b^
Within-subject difference (I-P) (VAS15)		−4.2 ± 20.6	−20.4 ± 21.7	5.4 ± 12.7	0.0011^b^
	^a^Mean ± SD; n (%)				
	^b^Welch’s t-test				
	^c^Fisher’s exact test				

Univariate analysis of responder status revealed statistically significant differences in age and sex (age: p = 0.0044, sex: p = 0.0132). The means and SDs of baseline VAS scores were 56.4 ± 23.4 for women (n = 23) and 27.8 ± 14.6 for men (n = 12).

A stepwise analysis was conducted using variables such as age, sex, and baseline VAS score (mean of I and P), each of which had a p-value of <0.2 in the univariate analysis shown in Table 1. We initially fitted a logistic regression model with age, sex, and baseline VAS score as explanatory variables; however, baseline VAS score did not predict responder status (Table 2).

**Table 2:** Results of multivariate logistic regression analysis. AIC: Akaike’s information criterion; VAS: visual analog scale; I: ibuprofen; P: placebo.

	Estimation of odds ratios (n = 35, AIC = 41.525)				
	Reference	Unit	Odds ratio	95% Confidence interval	p-value
Sex (female)	Male		11.43	1.153-287.199	0.0365
Age		−10 years	4.143	1.178-31.490	0.0226
Baseline VAS (mean of I and P)		10 mm	0.956	0.641-1.403	0.8151

The AIC served as the criterion for model selection. Notable findings included an odds ratio of 4.163 (95% CI: 1.170-31.952, p = 0.0233) for age (per -10 years) and an odds ratio of 9.977 (95% CI: 1.336-208.256, p = 0.0213) for sex (female), as detailed in Table 3.

**Table 3:** Results of multivariate logistic regression analysis (final model). AIC: Akaike’s information criterion; VAS: visual analog scale; I: ibuprofen; P: placebo.

	Estimation of odds ratios (n = 35, AIC = 39.580)				
	Reference	Unit	Odds ratio	95% Confidence interval	p-value
Sex (female)	Male		9.977	1.366-208.256	0.0213
Age		−10 years	4.163	1.170-31.952	0.0233

## Discussion

To our knowledge, this study represents a pioneering effort to explore to examine the correlations of patient characteristics with ibuprofen gargle efficacy in improving pain after mandibular third molar extractions. Regarding the efficacy endpoint in our study, responders were female and younger than non-responders. The influence of the higher baseline VAS score for women was minimal, despite the large number of women who were responders. Consistent with existing literature, our findings suggest that women exhibit lower pain thresholds, and baseline pain levels in women appear to be higher. Additionally, systematic reviews have found that women are more likely to respond to pain treatment [27]. Our data support the notion that the efficacy of NSAIDs in postoperative pain management varies according to age and sex; the results of some studies have suggested that naproxen alone could manage acute postoperative pain more effectively in women than in men [28]. The results of the present study suggest that women are more likely to benefit from the short-term analgesic effects of ibuprofen gargle after mandibular third molar extraction. Generally, local NSAID therapies do not differ in efficacy according to age, but there are differences in analgesic efficacy among NSAIDs [29]. The results highlight rapid recovery and better quality of life in patients aged <21 years compared with older patients [30]. Thus, age-related variation in the effect of ibuprofen-containing gargles may have been influenced by differences in recovery after third molar extraction.

In addition to our current findings regarding gargle treatment that future research will explore, we are currently planning to conduct a clinical trial (https://jrct.niph.go.jp/latest-detail/jRCTs051230162) in which cyclooxygenase-2 paste will be directly applied to the extraction socket for local pain control; such an approach may be required for non-responder patients, such as men.

Although this study represents a substantial exploratory advance of the differential effects of ibuprofen gargle on postoperative pain relief after mandibular third molar extractions, it also had limitations that influence its implications for future research. The methodology used in this study provides a robust framework for assessing the immediate analgesic effects of ibuprofen gargle, offering valuable insights into patient-specific responses according to baseline characteristics. However, the findings were derived from a relatively small cohort, and the statistical analysis was primarily descriptive. Although this limitation may impact the external validity, the results of this study establish a foundation for future meta-analyses and highlight areas that warrant further investigation. Moreover, the study's assessment of pain at only two-time points (five minutes and 15 minutes post-treatment) may not have fully captured the dynamic nature of postoperative pain management. The absence of well-established objective markers for evaluating the analgesic effects of ibuprofen gargles underscores the need for continued research in this area. By addressing these issues, we aim to provide a comprehensive perspective regarding our study's contributions to the field and to provide clear directions for future research endeavors.

## Conclusions

Based on data from a well-conducted clinical trial, this study showed that patient characteristics are associated with ibuprofen gargle efficacy in improving pain among patients who have undergone mandibular third molar extractions. Our results suggest that patients who are female and young tend to show greater improvement in pain control. Further studies are required to evaluate the relationship between the analgesic effect and baseline patient characteristics.
